# Involvement of the Nucleus Accumbens in Chocolate-induced Cataplexy

**DOI:** 10.1038/s41598-020-61823-4

**Published:** 2020-03-18

**Authors:** Jingyang Su, Zhi Li, Akira Yamashita, Ikue Kusumoto-Yoshida, Takuto Isomichi, Liying Hao, Tomoyuki Kuwaki

**Affiliations:** 1 Department of Physiology, Graduate School of Medical and Dental Sciences, Kagoshima University, Kagoshima, Japan; 2 Department of Pharmaceutical Toxicology, School of Pharmacy, China Medical University, Shenyang, China

**Keywords:** Emotion, Neurophysiology

## Abstract

Happiness is key for both mental and physical well-being. To further understand the brain mechanisms involved, we utilized the cataplexy that occurs in narcoleptic animal models as a quantitative behavioral measure because it is triggered by actions associated with happiness, such as laughter in humans and palatable foods in mice. Here we report that the rostral part of the nucleus accumbens (NAc) shell is strongly activated during the beginning of chocolate-induced cataplexy in orexin neuron-ablated mice. We made a local lesion in the NAc using ibotenic acid and observed the animals’ behavior. The number of cataplexy bouts was negatively correlated to the lesion size. We also examined the hedonic response to palatable food by measuring the number of tongue protrusions in response to presentation of honey, which was also found to be negatively correlated to the lesion size. Next, we used clozapine N-oxide to either activate or inactivate the NAc through viral DREADD expression. As expected, the number of cataplexy bouts increased with activation and decreased with inactivation, and saline control injections showed no changes. Hedonic response in the DREADD experiment varied and showed both increases and decreases across mice. These results demonstrated that the rostral part of the NAc plays a crucial role in triggering cataplexy and hedonic orofacial movements. Since the NAc is also implicated in motivated behavior, we propose that the NAc is one of the key brain structures involved in happiness and is a driving force for positive emotion-related behaviors.

## Introduction

Happiness is a key emotion for both mental and physical well-being. Although its benefits are well known, the underlying brain mechanisms are not well understood. Brain imaging studies in humans^1^ predicted the possible involvement of several brain structures in happiness, such as the nucleus accumbens (NAc), the ventral pallidum, and the anterior cingulate cortex. However, more detailed information on the neurotransmitters and neuronal pathways involved in the interaction between emotion and bodily health is currently unavailable. In experimental animals, only limited reports are available presumably due to a shortage of reliable and quantitative measures of behaviors associated with happiness. One exception are the reports from the Berridge group that used facial expressions such as tongue protrusions for an animal model of happiness and “liking”^2^. However, measuring only tongue protrusions is insufficient for evaluating possible happiness experienced from sources other than food. If multiple reliable animal models for enjoyment or happiness are developed, a more solid idea of the neuronal mechanisms involved can be elucidated.

We used the cataplexy that occurs in animal models of narcolepsy as an alternate quantitative animal model for happiness because cataplexy is triggered during behaviors associated with positive feelings, such as laughter in humans^3^ and palatable foods in mice^4^. The brain region responsible for cataplexy in orexin knockout mice, an animal model of narcolepsy^5^, has already been reported to be the medial prefrontal cortex^6^. Although this report showed an overall correlation between the activity of the medial prefrontal cortex via c-Fos expression and the frequency of cataplexy during the observation period, the question of whether the relationship is immediate and one-to-one remained. In the present study, we examined a closer relationship between cataplexy and activated brain sites. We used another activation marker, the phosphorylated form of extracellular signal-regulated kinase (pERK), because it is induced more quickly and with a smaller time window than c-Fos^7,8^. We used orexin neuron-ablated (ORX-AB) mice, another animal model of human narcolepsy, rather than prepro-orexin knockout mice^5^ because they show more bouts of cataplexy^9^ and their loss of orexinergic cells more closely resembles human narcolepsy^10^. The brain was taken directly following a cataplexy attack to enable the examination of a shorter time scale between the cataplexy and any potentially activated brain sites. Comparison between animals that experienced chocolate-induced cataplexy and those that ate chocolate but did not experience cataplexy allowed us to obtain information specific to cataplexy and not some other potentially unrelated action.

We found that the rostral part of the nucleus accumbens (NAc) was strongly activated at the beginning of cataplexy (see Results section). The NAc is a region in the basal forebrain rostral to the preoptic area of the hypothalamus. Various neuronal subtypes within the NAc core and shell are responsible for different cognitive functions^11,12^. Several roles are attributed to the NAc, such as the control of motivation, aversion, incentive salience, pleasure, positive reinforcement, response to stress, and regulation of depressive behavior^2,13–19^. Of these, we paid special attention to the reports from Berridge’s group^2^ showing that this region is one of the hedonic hot spots in the brain. We hypothesized that the NAc would be important for happiness and thus responsible for inducing cataplexy and tongue protrusions in response to palatable food. To test our hypothesis, we first used a neurotoxin, ibotenic acid, to lesion the NAc neurons in mice and observed the resulting behaviors. Next, we used designer receptors exclusively activated by designer drugs (DREADDs) to activate or inactivate the NAc. We used adeno associated virus (AAV) as a vector to express hM3Dq-mCherry or hM4Di-mCherry under the control of calmodulin kinase IIα (CaMKIIα) promoter.

## Results

### Chocolate-induced cataplexy was associated with immediate increase in pERK in the rostral part of the NAc

**Figure 1:**
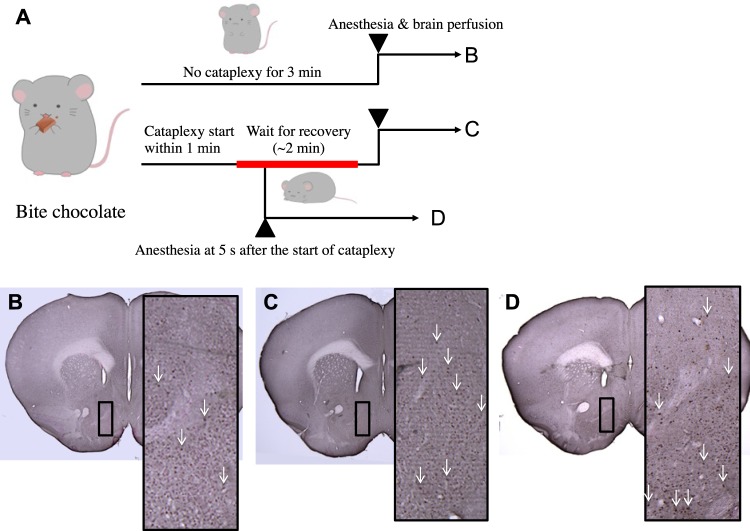
pERK immunostaining in rostral part of the NAc. (**A**) Schedule of brain sampling. Typical photograph taken from a brain in (**B**) no cataplexy group, (**C**) end of cataplexy group, and (**D**) start of cataplexy group. Both large and small rectangles denote the counting area (400 × 1000 µm) in the NAc shell. Arrows indicate positive immunoreactivity. Drawings in (**A**) were kindly provided by Ms. Jun Kaminosono.

Overall, we examined activation in 33 brain areas. We included the areas reported to be related to cataplexy as revealed by c-Fos staining^6^, but we focused on immediate activation of neurons rather than activation occurring over the course of hours by examining pERK immunoreactivity. The brains of mice who experienced chocolate-induced cataplexy and the brains of mice who ingested chocolate but did not experience cataplexy were compared. Two-way (sampling timing x brain regions) ANOVA revealed a significant difference among sampling timing (F2, 330 = 15.90, P < 0.0001), brain region (F32, 330 = 41.36, P < 0.0001), and interaction (F64, 330 = 2.421, P < 0.0001). With a multiple comparison test, we found that activation of 4 brain regions (rostral part of the NAc, 231% of the control, t = 6.532, P < 0.0001; caudal part of the NAc, 144%, t = 3.819, P = 0.0105; bed nucleus of the stria terminalis, 152%, t = 4.911, P < 0.0001; the dorsomedial hypothalamus, t = 3.619, P = 0.0223; Table 1 and Fig. 1) were associated with the occurrence of cataplexy. Cataplexy attacks lasted 116 ± 24 s (n = 5). To minimize the possible effect of cataplexy on neuronal activity and to address possible triggers for inducing cataplexy, we next anesthetized mice 5 s after the start of cataplexy. In this condition, the density of pERK was significantly larger only in the rostral part of the NAc (205% of the control group, t = 4.515, P = 0.0006, the right most column in Table 1 and Fig. 1D). This result indicated that activation of the rostral part of the NAc was critical in triggering cataplexy but not a consequence of chocolate ingestion or the cataplexy attack itself.

**Table 1:** Expression of pERK in orexin neuron-ablated mice after ingestion of chocolate.

Area	Without cataplexy (n = 5)	With cataplexy	
		End of cataplexy (n = 5)	Start of cataplexy (n = 3)
Anterior cingulate cortex	13.3 ± 1.1	16.5 ± 1.5	11.8 ± 1.2
Prelimbic cortex	13.9 ± 1.9	16.3 ± 1.4	15.9 ± 1.9
Infralimbic cortex	13.4 ± 1.5	17.5 ± 1.1	14.1 ± 2.4
Orbitofrontal cortex	15.1 ± 2.1	16.7 ± 1.4	12.3 ± 5.8
Piriform cortex	6.5 ± 0.6	8.8 ± 0.5	4.2 ± 2.6
Nucleus accumbens, rostral	14.7 ± 1.8	33.9 ± 2.0***	30.1 ± 1.9***
Nucleus accumbens, caudal	25.5 ± 1.7	36.7 ± 2.7*	25.1 ± 3.5
Claustrum	16.7 ± 3.3	17.5 ± 0.9	18.0 ± 3.1
Agranular insular cortex	11.4 ± 0.9	12.0 ± 1.0	9.6 ± 1.5
Bed nucleus of stria terminalis	27.7 ± 2.9	42.2 ± 3.8***	38.8 ± 8.2
Ventral pallidum, rostral	8.9 ± 2.3	11.7 ± 1.8	4.3 ± 1.6
Ventral pallidum, caudal	8.6 ± 2.1	9.4 ± 2.6	13.7 ± 3.6
Rhomboid thalamic nucleus	14.2 ± 0.9	18.1 ± 2.3	18.0 ± 0.9
Paraventricular hypothalamic nucleus	29.9 ± 1.6	31.7 ± 3.6	26.2 ± 6.7
Habenular nucleus	8.7 ± 1.6	8.1 ± 1.3	11.2 ± 1.1
Paraventricular thalamic nucleus	19.9 ± 4.5	29.6 ± 3.8	31.2 ± 4.3
Basolateral nucleus of amygdala	9.3 ± 1.1	7.3 ± 1.8	5.5 ± 1.3
Central nucleus of amygdala	7.6 ± 1.3	8.2 ± 0.9	10.1 ± 1.3
Anterior cortical nucleus of amygdala	11.3 ± 2.4	11.9 ± 0.9	13.1 ± 5.7
Posterior basomedial nucleus of amygdala	4.9 ± 0.9	5.2 ± 0.3	6.7 ± 1.1
Perirhinal cortex	7.3 ± 1.3	8.3 ± 1.2	7.4 ± 1.0
Dorsomedial hypothalamic nucleus	12.1 ± 1.5	22.8 ± 1.9*	16.5 ± 0.2
Lateral hypothalamus	9.2 ± 1.8	10.8 ± 0.9	8.7 ± 0.7
Subparafascicular thalamic nucleus	5.7 ± 0.9	7.3 ± 0.6	4.5 ± 2.4
Posterior hypothalamus	14.5 ± 2.0	18.8 ± 2.5	15.0 ± 1.9
Parasubthalamic nucleus	12.7 ± 1.8	13.0 ± 2.4	14.3 ± 3.7
Tuberomammillary nucleus	15.0 ± 1.2	18.3 ± 1.8	13.2 ± 1.0
Periaqueductal gray	3.3 ± 0.8	7.1 ± 1.3	5.1 ± 0.6
Anterior pretectal nucleus	4.0 ± 0.8	5.3 ± 1.6	3.6 ± 1.7
Edinger-Westphal nucleus	26.1 ± 3.9	16.0 ± 2.5*	29.3 ± 2.7
Ventral tegmental area	7.0 ± 1.5	8.3 ± 0.5	7.6 ± 0.4
Locus coeruleus	38.0 ± 3.4	31.1 ± 2.0	26.2 ± 5.9*
Nucleus of the solitary tract	17.8 ± 0.9	22.2 ± 2.1	21.9 ± 2.1

Values were the number of pERK positive cells per unit area of 0.1 mm^2^ and expressed as mean ± SEM. *p < 0.05, **p < 0.01, ***p < 0.001 vs. without cataplexy control (Sidak post hoc test).

### Number of cataplexy bouts and number of tongue protrusions were decreased by lesioning the NAc

**Figure 2:**
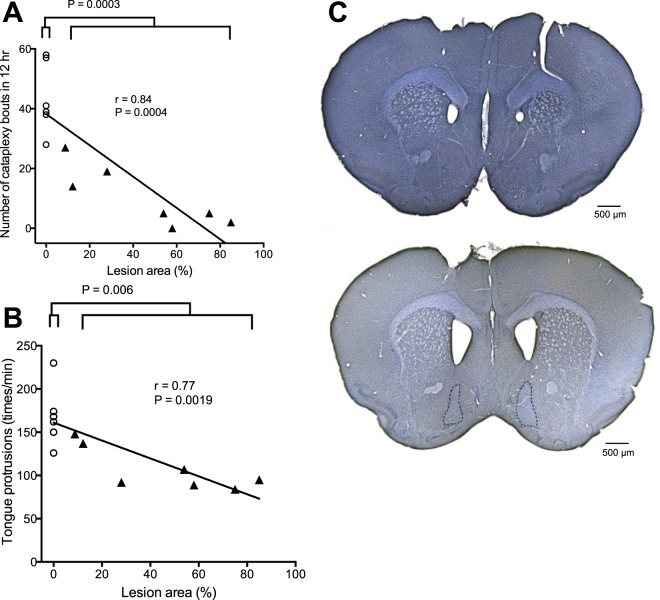
Lesioning of the NAc with ibotenic acid reduced cataplexy and tongue protrusions. (**A**) The number of cataplexy bouts during the observation period of 12 hr was negatively correlated with the lesion size. Comparison of the group average between the PBS group (circles, n = 6) and ibotenic acid group (triangles, n = 7) revealed a significant difference (P = 0.0003, t-test) in the number of cataplexy bouts. (**B**) In the same animals as A, the number of tongue protrusions in response to honey was also negatively correlated to the lesion size. Again, comparison of the group average between the PBS group (circles, n = 6) and ibotenic acid group (triangles, n = 7) revealed a significant difference (P = 0.006, t-test) in the number of tongue protrusions. (**C**) Lesion size was estimated as the area with no NeuN staining. Upper and lower images were taken from an animal with PBS injection and ibotenic acid injection, respectively. Dotted line in the lower photograph denotes the border of the unstained area. Scale bar: 500 µm. In (**A**,**B**), the lesion size was normalized as the percentage of the total area of the rostral part of the NAc shell.

To examine a possible causal relationship between the activity of the NAc and the occurrence of cataplexy attacks, we microinjected a neurotoxin, ibotenic acid (n = 7), or PBS (n = 6) bilaterally into the NAc of ORX-AB mice and observed the frequency of the cataplexy bouts. When PBS was injected, unaltered NeuN staining indicated no lesioning occurred in the NAc (Fig. 2C, upper) and mice had 28–57 bouts of cataplexy during the 12 hr of observation (Fig. 2A, open circles). In the ibotenic acid group, the lesion size varied from 8.8% to 85.0% of the total area of the rostral part of the NAc shell (Fig. 2A,C, lower). Mice with bilateral lesions that covered more than 50% of the total area showed less than 5 bouts of cataplexy (Fig. 2A lower right triangles, n = 4), while mice with unilateral lesions less than 30% of the total area showed more than 14 bouts of cataplexy (Fig. 2A upper left triangles, n = 3). Nevertheless, the numbers of cataplexy bouts in the ibotenic acid group (10.3 ± 3.8, n = 7) was significantly lower than in the PBS group (43.5 ± 4.8, n = 6, t = 5.442, P = 0.0003, Student’s t-test). In addition, correlation analysis using data from both the ibotenic acid and PBS groups (n = 13) revealed that the regression coefficient was 0.84 and was significantly different from 0 (F1,11 = 25.56, P = 0.0004), indicating a negative relationship between the lesion size and the number of cataplexy bouts. Lesioning of the NAc did not affect locomotor activity (5.2 ± 0.7 arbitrary unit in the PBS group vs. 4.0 ± 0.6 in the ibotenic acid group, t = 1.302, P = 0.219) or amount of chocolate ingested (1.34 ± 0.06 g in the PBS group vs. 1.34 ± 0.03 in the ibotenic acid group, t = 0.01937, P = 0.985) during the 12 hr of observation.

The number of tongue protrusions was negatively correlated (r = 0.77, F1, 11 = 16.54, P = 0.0019, n = 13) to the lesion size similarly to cataplexy (Fig. 2B). It should be pointed out, however, that the animals with large lesions (>50%, n = 4) demonstrated almost a complete absence of cataplexy (3.0 ± 1.2 times vs. 43.8 ± 5.7 times in PBS group, 93% decrease) while the same animals showed only a moderate decrease in tongue protrusions (94 ± 5 times vs. 168 ± 14 times in PBS group, 44% decrease).

### The number of cataplexy bouts was increased by AAV-Gq and decreased by AAV-Gi

**Figure 3:**
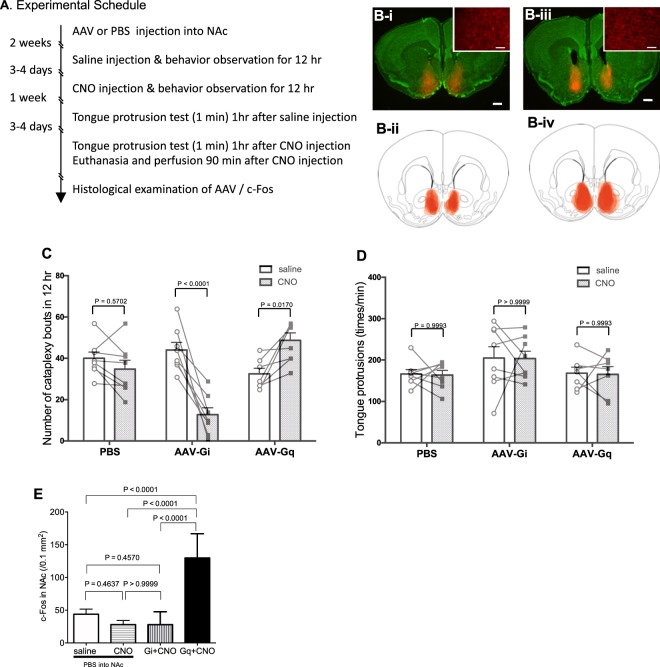
Effect of activation/inactivation of the rostral part of the NAc shell on cataplexy and tongue protrusions. (**A**) Experimental schedule. Each animal went through 4 behavioral measurements: Cataplexy observation after saline injection and CNO injection, and the tongue protrusion test after saline injection and CNO injection. Finally, animals were euthanized after another injection of CNO. (**B**) B-i and B-iii show typical examples of AAV spread (red mCherry signal) from an AAV-Gi injected mouse and an AAV-Gq injected mouse, respectively. Sections were counterstained with Neurotrace 500/525 green fluorescent Nissl stain for better detection of the region border. Scale bar: 500 µm. Inset in B-i and B-iii show higher magnification of typical examples of AAV-Gi and AAV-Gq signal, respectively, to show cellular distribution of mCherry. Scale bar: 50 µm. B-ii and B-iv show estimated infection areas overlaid in a single diagram taken from the mouse brain atlas^49^ from 8 Gi animals and 7 Gq animals, respectively. (**C**) Observation of cataplexy bouts. (**D**) Result from the tongue protrusion test. In (**C**,**D**), statistical results using Sidak’s multiple comparison test are shown (see text for results of the preceding 2-way ANOVA). Used number of animals were n = 8 for PBS injected group, n = 8 for AAV-Gi injected group, and n = 7 for AAV-Gq injected group. (**E**) Activity of the rostral part of the NAc shell was estimated by c-Fos immunoreactivity. In this case, we used 8 additional animals that were injected with PBS into the NAc and with saline i.p. before being euthanized. The number of c-Fos positive cells was counted within a rectangle (400 × 1000 µm) drawn in the NAc as was indicated in Fig. 1. Statistical results using Tukey’s multiple comparison test are shown (see text for results of the preceding 1-way ANOVA).

We next examined the possible effect of activation/inactivation of NAc on cataplexy and tongue protrusions using the DREADD system (Fig. 3A). In this experiment, AAV-Gi/Gq was injected into the NAc (Fig. 3B). Successful injections showed mCherry expression in NAc neurons (inset in Fig. 3B-i,B-iii) and the overall distribution of mCherry was centered on and overlapped with the rostral NAc shell region (Fig. 3B-ii,B-iv). We found 338 ± 45 (mean ± SE, n = 8) mCherry positive cells in the AAV-Gi group and 257 ± 25 (mean ± SE, n = 7) in the AAV-Gq group. These values corresponded to 84.6 ± 11.1 cells in the Gi group and 64.2 ± 6.2 in the Gq group within the counting area of 0.1 mm^2^ and were greater than the number of pERK positive cells at the start of cataplexy (Table 1). No apparent localization within the NAc of mCherry positive cells was found.

When the cataplexy bouts among the 6 groups were compared (pretreatment with AAV-Gi, AAV-Gq, and PBS x injection of saline or CNO, Fig. 3C) using a two-way ANOVA, there was significant difference among pretreatment (F2, 20 = 4.675, P = 0.0216), injected drug (F1, 20 = 8.715, P = 0.0079), and interaction (F2, 20 = 26.76, P < 0.0001). The multiple comparison test revealed there was no difference between the saline injected AAV-Gi group (44.0 ± 3.7 times/12 hr, n = 8) and the PBS group (40.0 ± 3.0, n = 8, t = 0.8251, P = 0.7990), between the saline AAV-Gq group (32.4 ± 2.7, n = 7) and the PBS group (t = 1.509, P = 0.3622), and between the saline AAV-Gi group and the saline AAV-Gq group (t = 2.306, P = 0.0771). In addition, these values were very similar to those in experiment 2 (43.5 ± 4.8 times/12 hr) where saline was not injected before the observation period (Fig. 2A). These results show that neither the AAV infection of the NAc itself and the resulting expression of Gi/Gq nor the intraperitoneal injection of saline at the start of recording session affected the number of cataplexy bouts during the 12 hr observation period. When CNO was injected, the resulting change in the number of cataplexy bouts in the Gq group were opposite of the Gi group. CNO induced a significant increase in the Gq group (from 32.4 ± 2.7 to 46.6 ± 3.6, t = 3.097, P = 0.0170, Sidak’s multiple comparison test) while it induced significant decrease in the Gi group (from 44.0 ± 5.4 to 12.8 ± 3.3, t = 7.315, P < 0.0001), as was the case in ibotenic acid lesioning. In the PBS pretreated group, CNO did not change the number of cataplexy bouts (from 40.0 ± 3.0 to 34.8 ± 4.3, t = 1.229, P = 0.5494).

In the Gi group, CNO did not affect locomotor activity (4.7 ± 1.1 arbitrary unit after saline vs. 2.9 ± 0.8 after CNO, n = 8, t = 2.019, P = 0.083, paired t-test) or amount of chocolate ingested (1.44 ± 0.10 g after saline vs. 1.58 ± 0.12 after CNO, t = 1.553, P = 0.164), as was the case in the ibotenic acid experiment. In the Gq group, CNO did not affect locomotor activity (4.6 ± 1.3 arbitrary unit after saline was injected vs. 6.9 ± 1.1 after CNO, n = 7, t = 1.697, P = 0.141, paired t-test) or amount of chocolate ingested (1.70 ± 0.18 g after saline vs. 1.50 ± 0.18 after CNO, t = 1.183, P = 0.275).

When the number of tongue protrusions were compared among the 6 groups (pretreatment with AAV-Gi, AAV-Gq, and PBS x injection of saline or CNO) (Fig. 3D) using two-way ANOVA, there was no significant difference among pretreatment (F2, 20 = 2.806, P = 0.0843), injected drug (F1, 20 = 0.02647, P = 0.8724), and interaction (F2, 20 = 0.001258, P = 0.9987). The multiple comparison test revealed no difference between the saline injected AAV-Gi group (205 ± 27 times/min, n = 8) and the PBS group (166 ± 11, n = 8, t = 1.568, P = 0.3295), between the saline AAV-Gq group (168 ± 15, n = 7) and PBS group (t = 0.07448, P = 0.9998), and between the saline AAV-Gi group and the saline AAV-Gq group (t = 1.440, P = 0.4021). In addition, these values were also very similar to those in experiment 2 (168 ± 17, Fig. 2B) where saline was not injected before the tongue protrusion test, indicating that AAV infection of the NAc and the resulting expression of Gi/Gq or intraperitoneal injection of saline 60 min prior to the test did not affect the number of basal tongue protrusions in response to honey. Contrary to our expectations, however, activation or inactivation of the NAc by Gi/Gq did not consistently change the number of tongue protrusions. In the Gi group, the number of tongue protrusions decreased in only 4 of 8 animals whereas cataplexy bouts decreased in all 8 animals (Fig. 3C,D). In the Gq group, the number of tongue protrusions increased in only 3 of 7 animals whereas cataplexy bouts increased in 7 of 7 animals.

Cellular activity in the NAc induced by CNO was estimated by counting c-Fos positive cells in the NAc (Fig. 3E). One-way ANOVA revealed there was a significant difference (F3, 27 = 36.51, P < 0.0001) among 4 groups (PBS + saline, PBS + CNO, Gi + CNO, Gq + CNO). As expected, CNO induced significantly more c-Fos positive cells in the Gq + CNO group than in the PBS + saline (P < 0.0001, 1-way ANOVA followed by Tukey’s multiple comparison test) and the PBS + CNO group (P < 0.0001). Unexpectedly, CNO induced no significant change in c-Fos in the Gi group when compared to the PBS + saline group (P = 0.4722) and the PBS + CNO group (P > 0.9999).

## Discussion

### Activity of the NAc was closely related to the triggering of cataplexy

In this study, we found that the rostral part of the NAc was strongly activated at the beginning of cataplexy in ORX-AB mice (Table 1). Other brain areas, including the medial prefrontal cortex (the anterior cingulate cortex, the prelimbic cortex, and the infralimbic cortex), were not. These results are a clear contrast to a previous report^6^ which showed a strong activation of the medial prefrontal cortex and scarce activation of the NAc. Although there are some discrepancies between the results, there are possible explanations that may account for the differences found in our study. First, we focused on the beginning of cataplexy in hopes of finding the region responsible for inducing it. The previous study allowed animals 3 hr to ingest chocolate and have up to 30 bouts of cataplexy. Therefore, activation of the medial prefrontal cortex may be indicative of both the cause and result of multiple cataplexy bouts. Second, we compared chocolate-induced cataplexy to chocolate ingestion that did not result in cataplexy. As a result, any possible effects of chocolate intake would be equivalent in both groups. In contrast, the previous study compared a chocolate group with a regular chow group, so the results shown may be from the effects of chocolate ingestion itself in addition to the number of cataplexy bouts. Third, we used pERK and the previous report used c-Fos as the activation marker. Different activation markers have different temporal resolution^7^ and are expressed following different intracellular events^20^ which may account for the different activation patterns. Fourth, we used ORX-AB mice and the previous study used orexin knockout mice. Ablation of orexin neurons may induce secondary changes in neuronal populations that are closely linked with them and/or eliminates neurotransmitters and neuromodulators that are co-expressed and may cooperate with orexin, such as glutamate and dynorphin^21^, so cataplexy can no longer be modulated as effectively. Induction of secondary changes in other populations is unlikely because no such evidence was obtained during anatomical inspection of the region. For example, distribution of melatonin concentrating hormone (MCH)-producing neurons remained normal even though their location is intermingled among the orexin neuronal population in the hypothalamus^9,22^. However, while the importance of the elimination of co-expressed transmitters cannot be dismissed because ORX-AB mice show cataplexy more frequently than orexin knockout mice^9^, there is no report showing a qualitative difference in the cataplexy that occurs between them. Therefore, it is difficult to determine if the discrepancy in the results between the previous study (prefrontal cortex in orexin knockout mice) and the current study (NAc in ORX-AB mice) is caused by the different animal models used. Taking these aspects into consideration, we think our results are compatible with the previous report. Interestingly, there are reciprocal connections between the medial prefrontal cortex and the NAc^23^ and information from either site may depend on the other before triggering cataplexy.

### The NAc is required for both cataplexy and tongue protrusion

To examine the possible role of the NAc in chocolate-induced cataplexy, we first lesioned the NAc via microinjection of ibotenic acid. The resulting lesion size varied from animal to animal and enabled us to analyze the correlation between lesion size and animal behavior. A significant negative relationship between the lesion size and the number of cataplexy bouts (Fig. 2A) and number of tongue protrusions (Fig. 2B) was observed, indicating the NAc is required for both behaviors. However, there was a quantitative difference between cataplexy bouts and tongue protrusions. Lesions that caused over 50% loss in bilateral NAc resulted in near complete loss of cataplexy but the same animals showed only ~40% loss in tongue protrusions. The latter observation is in line with a previous report^24^. Therefore, “liking” behavior and happiness seemed to depend on not only the NAc but other brain areas as well.

### Technical consideration of CNO use

Another previous report showed that clozapine, a conversion product of CNO *in vivo*^25^, induces cataplexy-like negative myoclonus in schizophrenia patients as a rare side effect of clozapine^26,27^. In control mice that were pre-treated with PBS, CNO did not change the number of cataplexy bouts or tongue protrusions (Fig. 3C,D) when compared to saline when injected into the same animal. CNO dosing in this study was far lower than that which might induce cataplexy-like behavior. We used 0.45 mg/kg of CNO in our experiment so the equivalent dose of clozapine would be 0.005 mg/kg because the clozapine dose required to induce behavioral change *in vivo* through hM3Dq was 100 times more potent than CNO^25^. The clinical dose of clozapine is 12.5–600 mg/day^28^, which corresponds to 0.2–10 mg/kg when assuming a human body weight of 60 kg. Therefore, the dosing of CNO in this study was far lower (1/40–1/2000) than that which might induce cataplexy-like behavior.

### The NAc is sufficient for triggering cataplexy

The area of AAV infection, as estimated from the distribution of mCherry, was similar between AAV-Gi (Fig. 3B-i,ii) and AAV-Gq (Fig. 3B-iii,iv) and overlapped with the rostral part of the NAc. Increasing the volume of injection from 50 to 300 nl allowed for a more consistent coverage of a wider area of the NAc when compared to the ibotenic acid experiments. Nevertheless, infection of both AAV-Gq and AAV-Gi did not affect the basal numbers of cataplexy bouts (compare saline columns in Fig. 3C) and tongue protrusions (Fig. 3D), indicating AAV infection or expression of artificial receptors themselves did not affect basal activity in NAc neurons. In addition, neither locomotor activity nor intake of chocolate changed after the administration of CNO. Thus, the decreased number of cataplexy bouts by the administration of CNO in AAV-Gi mice (Fig. 3C, middle) and increased number in AAV-Gq mice (Fig. 3C, right) seemed specifically related to the role of the NAc in triggering cataplexy.

CNO treatment induced a significantly larger number of c-Fos expressing cells in the NAc of AAV-Gq mice than in the PBS mice (Fig. 3E). This result indicated that CNO had the potential to activate the NAc in AAV-Gq mice. However, the exact relationship between CNO-induced activation and the number of cataplexy bouts could not be inferred because behavior and c-Fos were measured on different days (Fig. 3A). CNO treatment did not significantly affect c-Fos numbers in the AAV-Gi mice. Although the exact reason is unclear, basal expression of c-Fos-positive cells was so small that it was difficult to detect a decrease. Another possibility is that c-Fos was not a suitable indicator for assessing the level of activity in the NAc during cataplexy induction. In either case, however, we think c-Fos was a more suitable marker than pERK to examine the possible effect of CNO because expression of pERK is transient^7^ and as a result we were unable to distinguish between CNO-induced cataplexy and cataplexy caused by other sources. Since manipulation of NAc activity resulted in bidirectional changes in the number of cataplexy bouts, it was not only required but also sufficient to trigger cataplexy. Inhibition of the medial prefrontal cortex^6^ or the amygdala^4,29^ decreased the number of cataplexy bouts, and activation of the amygdala^29,30^ increased the number of cataplexy bouts. Thus, the amygdala also seems to be required and is sufficient to induce cataplexy on its own, although it was not activated during the beginning of cataplexy in this study (Table 1).

### Is cataplexy useful as an animal model of happiness?

One report showed that cataplexy attacks could be increased (by ~100%) by aversive predator odor in orexin knockout mice^31^. Thus, cataplexy attacks may not always be associated with positive emotions. However, human cataplexy episodes are mainly triggered by positive emotions, especially laughter^3^, and a dramatic increase (>700%) was observed when giving chocolate to mice^6^. In this study, we used chocolate to induce cataplexy and, as a result, all the cataplexy reported here should be attributed to only positive emotions even though it is difficult to determine exactly what “happiness” looks like in mice. Although we used chocolate as a trigger to induce cataplexy in this study and orexin is implicated in increasing appetite^32^, we do not think that the NAc directly regulates appetite for several reasons. First, in both AAV-Gq and AAV-Gi mice, CNO changed the number of cataplexy bouts but did not change the amount of chocolate and food consumed. Second, in experiment 1 (pERK study), we compared chocolate-induced cataplexy to chocolate ingestion without cataplexy. As a result, any possible differences are likely unrelated to chocolate ingestion. Third, we used ORX-AB mice for all experiments so any orexin-related appetite effects would not occur. Nevertheless, possible interference from the food-related motivational function of the NAc cannot be ignored. To further examine this possibility, using positive emotion-triggering stimuli other than food, such as playing with toys and contact with potential mates, is necessary. In this context, however, evaluating cataplexy does seem to be a promising method to explore positive emotion.

Although “liking” behavior did not change in the same way as cataplexy through the manipulation of NAc activity in this study, there are some explanations as to why this may be the case. Some psychologists divide positive emotions into subcategories such as happy, pleasure, excitement, and satisfaction^33^. Cataplexy-inducing emotions and “liking” emotions may belong to different subcategories, although imaging studies reveal a striking similarity in the active sites for different categories of positive emotions^1^. A closer look at the NAc revealed two types of GABAergic output neurons, or medium spiny neurons (MSN), are known: one population contains enkephalin and dopamine D2 receptors and projects to the pallidum, and the other contains substance P and dopamine D1 receptors and projects to the substantia nigra^34^. It is not known at present which neurons are related to cataplexy-inducing emotion but distribution of CaMKII is restricted to D1-type MSN^35^ and “liking” is related to enkephalin-containing (presumably D2-type) cells^2^. This may explain why ibotenic acid lesioning decreased both cataplexy and “liking” whereas AAV-CaMKII-Gi only inhibited cataplexy. Another explanation for why the behavioral changes in the ibotenic acid group were more severe than those in the AAV-CaMKII-Gi group could be that the lesioning damaged not only cell bodies but also fibers of passage^36^, contrary to the original reporting of it being an axon-sparing lesioning technique^37^. Therefore, the decreased “liking” behavior in the lesioning portion of this study may not be solely accounted for by loss in the NAc. Of course, a closer examination of the chemical identity of cataplexy-related neurons is necessary before making any conclusive statements. Another possibility was the methodological difference. We observed cataplexy for 12 hr while we observed “liking” behavior for only 1 min. If we observed “liking” behavior for a longer time, similar alterations to those in cataplexy might be observed. We believe that measuring cataplexy is a promising addition to measuring “liking” behavior for the purpose of studying positive emotion in narcoleptic animals. However, we need more methodologies with which to study happiness in normal animals because they do not show cataplexy. The NAc should be studied more extensively in the context of a mediator for positive emotions.

## Materials and Methods

### Ethics approval

All experiments were conducted at Kagoshima University in accordance with the guiding principles for the care and use of animals in the field of physiological sciences published by the Physiological Society of Japan (2015) and were approved by the Experimental Animal Research Committee of Kagoshima University (MD13075, MD17105).

### Animals

For the animal model of narcolepsy, we used 56 male orexin neuron-ablated (ORX-AB) mice^9,22^ that were 12–24 weeks old at the start of the experiment and weighed 24–36 g. The original mating pairs were a generous gift from Prof. Yamanaka at Nagoya University and were bred in Kagoshima University’s animal facility. We used ORX-AB mice rather than prepro-orexin knockout mice^5^, because the former shows more cataplexy than the latter^9^. A method for selective ablation of orexin neurons has previously been reported^9^. In short, orexin-tTA mice, which express tetracycline transactivator (tTA) exclusively in orexin neurons under the control of the human prepro-orexin promoter^9^, were bred with tetO diphtheria toxin A fragment (DTA) mice (B6.Cg-Tg (tetO DTA) 1Gfi/J, The Jackson Laboratory) to generate orexin-tTA; tetO DTA mice. In these double transgenic mice (called ORX-AB in this paper), doxycycline is removed from their chow starting from of birth so, by 4 months of age, almost all (>97%) of the orexin neurons are ablated^9^. Ablation of orexin neurons was confirmed in our previous studies^9,22^. In the current study, doxycycline was also removed chow starting from the date of birth and ablation of orexin neurons was confirmed (data not shown). All mice were housed in a room that was maintained at 22–24 °C with lights on at 19:00 and off at 7:00 for at least 1 month before experimentation started. We selected a reversed light/dark cycle so experimenters were able to observe mice in their active nocturnal phase of behavior during the daytime. We used male mice to avoid possible effects from an estrous cycle and housed them individually from start of the reversed light/dark cycle to avoid any possible social rank effect on male behavior^38^. In addition, female hormonal cycles, at least in humans, may have some effect on sleep-related disorders including narcolepsy with cataplexy^39^. Mice had food and water available *ad libitum*.

### Behavioral observation of cataplexy

On the experimental day, mice were individually placed into a recording chamber in a soundproof box with a 12:12 reversed light/dark cycle at 19:00 (lights on at 19:00). In addition to regular chow and water, we gave milk chocolate (one Hershey’s Kiss; Hershey) to induce positive emotion and increase the number of cataplexy attacks^6^. The chamber was illuminated with a far infrared lamp (940 nm, SA2-IR, World Musen, Hong Kong) during the dark period and with a visible light lamp during the light period. Mouse behavior was continuously recorded with a video camera (CBK21AF04, The Imaging Source Asia, Taipei, Taiwan) and monitored on a personal computer located outside the soundproof box using the video capture function in LabChart (ADInstruments Inc., Bella Vista, NSW, Australia). Locomotor activity was recorded with a passive pyroelectric infrared motion sensor (AMN 1111, Panasonic Co., Osaka, Japan) that was attached to the ceiling of the soundproof box. The pyroelectric infrared sensor generates a square analog pulse (3 V, 30 ms/detection) when it detects changes in infrared radiation^40^. The sensitivity of the movement sensor was set so that possible effects from ventilation would not be counted as movement. The sensor’s output signals were digitally converted at 100 Hz and transferred to a computer with PowerLab (ADInstruments) and were captured with LabChart along with the video recording. The video recording was performed over 24 hr (from 19:00 to 19:00 of the second day) of which the first half (light period) served as the acclimatization period because most cataplexy attacks observed in orexin deficient mice occur during the dark period^5,6,9^. Cataplexy was determined according to the established criteria for mice^41^ which is defined by several observable features. First is an abrupt episode of nuchal atonia lasting at least 10 s. Atonia was determined to be occurring when mice were in a prone position with their head and belly down in the bedding with their limbs and tail typically situated straight out from the trunk. This posture shows a clear contrast to a normal sleeping position in which mice are curled up and fold their limbs and tail underneath their trunk. Secondly, the mouse is immobile other than the movements associated with breathing during an episode. Finally, there must be at least 40 s of active wakefulness (moving) preceding the atonia episode.

### The tongue protrusion test

Castro and Berridge^42^ showed that pleasurable emotion in experimental animals can be judged by orofacial expressions and can be quantified by counting tongue protrusions (“liking” reactions) in response to the presentation of a sweet taste in rodents. Following their methods, we counted the number of tongue protrusions in response to a pure honey solution. A mouse was gently held in an experimenter’s hand and 0.1 ml of honey was slowly presented in front of the mouth using a syringe. The orofacial region was video recorded for 1 min and the number of tongue protrusions was counted with slow (1:2) playback using QuickTime v.7 software.

### Stereotaxic injection of ibotenic acid or AAV into NAc

Surgeries for injections were performed under isoflurane (3%, inhalation) anesthesia using a stereotaxic instrument (ST-7, Narishige, Tokyo, Japan). A glass micropipette (2-000-001, Drummond Scientific, Broomall, PA, USA) pulled with a puller (PC-10, Narishige) with a tip diameter of 10 μm was filled with ibotenic acid (0.32 M in PBS; 50 nl injected over 5 min), PBS (50 nl), recombinant adeno associated virus (AAV, serotype 5)-CaMKIIα-hM4Di-mCherry (Lot v4493, 4.4 × 10^12^ GC/ml, 300 nl/side over 10 min) or AAV(5)-CaMKIIα-hM3Dq-mCherry (Lot v9235, 3.1 × 10^12^ GC/ml, 300 nl/side over 10 min). These drugs were stereotaxically and bilaterally injected into the NAc of ORX-AB mice. A gas pressure injector system (IM-200J, Narishige) was connected to the glass micropipette and to a compressed nitrogen tank with a polyethylene tube. A pressure (5–10 kPa) was applied to inject the solution. Injection sites were as follows: anterior 1.42 mm, lateral ± 0.8 mm, ventral 4.75 mm from bregma. After pressure injection, the micropipette was left in place for 10 min before being slowly withdrawn. After surgery, mice were given an antibiotic (penicillin G, 40,000 U/kg) and an analgesic (buprenorphine, 0.05 mg/kg).

The majority of neurons in the NAc are GABAergic^43^ and early reports claimed that CaMKII is almost exclusively expressed in excitatory neurons with an exception being the olfactory bulb^44,45^. Nevertheless, we selected the CaMKIIα promoter because CaMKII is involved in phosphorylation of ERK^46^ and presence the of CaMKII in GABAergic neurons in the NAc has been reported^35,47,48^.

### Immunohistochemistry

Mice were deeply anesthetized with urethane (1.8 g/kg, i.p.) and transcardially perfused with 25 ml of phosphate-buffered saline (PBS, 0.01 M, pH 7.4), followed by 25 ml of 4% paraformaldehyde (PFA) solution. The brain was removed, post-fixed in 4% PFA solution at 4 °C overnight, and immersed in 30% sucrose in PBS at 4 °C for 2 days. A series of 40 μm sections was obtained with a vibratome (SuperMicroSlicer Zero1; DOSAKA EM, Kyoto, Japan) of which every fourth section was used for immunostaining. The brain sections were immersed in blocking solution (1% normal horse serum and 0.3% Triton-X in 0.01 M PBS) for 1 hr at room temperature. The sections were then incubated with primary antibodies overnight. Primary antibodies were diluted in blocking solution and the conditions were as follows: anti-phosphorylated form of extracellular signal-regulated kinase (pERK) rabbit antibody (4370 S, Cell Signaling Technology) at 1/400; anti-c-Fos rabbit antibody (ABE457, Millipore Corp.) at 1/1000; anti-NeuN guinea pig antibody (266004, Synaptic Systems) at 1/400. The sections were washed with PBS and then incubated with secondary antibody that was anti-rabbit IgG-biotin complex (711-065-152, Jackson Immunoresearch, 1/300) or anti-guinea pig IgG-biotin complex (706-065-148, Jackson Immunoresearch, 1/300) for 90 min. NeuN and pERK were processed with an ABC system (PK-7100, Vector Laboratories) for 90 min and visualized with DAB staining (SK-4100, Vector) with cobalt and nickel intensification. Finally, sections were counterstained with Neutral Red or Cresyl violet (conventional Nissl staining). For c-Fos staining in AAV experiments, the fluorescence method was used instead of the above conventional method because the AAV-Gq used also has the fluorescent marker, mCherry. Here, c-Fos was visualized with streptavidin-conjugated Alexa-488 (S11223, Invitrogen, 1/500). Sections in AAV experiment that were not stained for c-Fos were stained with Neurotrace 500/525 Green Fluorescent Nissl Stain (N21480, ThermoFisher Scientific) at 1/100 for 20 min to better contrast with mCherry.

### Quantitative analysis of histology

To determine the number of pERK positive cells, we counted nuclei using a counting box placed bilaterally over an area of interest. The counting box was placed on the regions reported by Oishi *et al*.^6^ with slight modification. For the anterior cingulate cortex, we placed a 500 × 350 µm (width x height) rectangular box on sections 1.98 mm rostral to bregma, with the dorsal edge of the box 700 µm below the dorsal-most point at which the hemispheres meet, and the medial border along the midline. We counted cells in the prelimbic cortex using an identical box placed just ventral to the anterior cingulate cortex box. For the infralimbic cortex, we placed an identical box just ventral to the prelimbic cortex box. For the orbitofrontal cortex, we placed a 1000 × 300 µm box just ventral to the infralimbic cortex box. For the piriform cortex, we placed a 1000 × 300 µm box with the lateral border along the rhinal fissure and the ventral border along the ventral brain surface 1.98 mm rostral to bregma. For the rostral part of the nucleus accumbens (NAc) shell, we placed a 400 × 1000 µm box just above the ventral pallidum VP and just lateral to the island of Calleja 1.42 mm rostral to bregma. For the caudal part of the NAc, we placed a 1000 × 400 µm box just above the ventral pallidum and just lateral to the major island of Calleja 0.98 mm rostral to bregma. For the agranular insular cortex, we placed a 400 × 500 µm box of which the dorsal edge was the ventral terminal of external capsule. For the claustrum, we placed a 250 × 500 µm box just medial to the agranular insular cortex box 0.50 mm rostral to bregma. For the bed nucleus of the stria terminalis, we placed a 250 × 500 µm box just below the lateral ventricle and just above the anterior commissure. For the rostral part the ventral pallidum, we placed a 45° angled 600 × 300 µm box of which the lateral edge was the piriform cortex and dorsal edge was the interstitial nucleus of the posterior limb of the anterior commissure 0.26 mm rostral to bregma. For the caudal part of the ventral pallidum, we placed a 45° angled 600 × 300 µm box just below the lateral globus pallidus 0.22 mm caudal from bregma. For the paraventricular hypothalamic nucleus (PVN), we placed a 250 × 400 µm rectangle just lateral to the dorsal edge of the third ventricle. For the rhomboid thalamic nuclei, we placed a 250 × 250 µm box 600 µm above the PVN rectangle 0.94 mm caudal from bregma. For the central nucleus of the amygdala (CeA), we placed a 45° angled 500 × 500 µm box just below the dorsal border of the optic tract. For the basolateral nucleus of the amygdala, we placed a 500 × 500 µm box with the ventral border along the ventral terminal of the external capsule and medial border along the lateral border of the CeA. For anterior cortical nucleus of the amygdala, we placed a 300 × 300 µm box 600 µm lateral from the ventral border of the optic tract. The habenular nucleus can be easily identified in Cresyl violet staining and a 250 × 400 µm oval box was placed on it. For the paraventricular thalamic nuclei, we placed a 300 × 300 µm box just below the third ventricle 1.46 mm caudal from bregma. For the posterior basomedial nucleus of the amygdala, we placed a 500 × 400 µm box with the dorsal lateral edge at the terminal of the external capsule. For the perirhinal cortex, we placed a 700 × 400 µm box with the ventral medial edge against the dorsal edge of the lateral nucleus of the amygdala. For the dorsomedial hypothalamic nucleus, we put a 600 × 600 µm box 600 µm above the ventral surface of the brain and the medial border along the midline. For the lateral hypothalamic area, we put a 600 × 600 µm box just lateral to the dorsomedial hypothalamic nucleus box on 1.94 mm caudal from bregma. For the tuberomammillary nucleus, we used a 200 × 200 µm box with the dorsal border at the dorsal edge of the third ventricle and the lateral border along the lateral surface of the hypothalamus. For the posterior hypothalamus, we put a 400 × 500 µm box just below the dorsal 3rd ventricle and the medial border along the midline. For the subparafascicular thalamic nucleus, we put a 600 × 400 µm box with the ventromedial corner of the box contacted the dorsolateral corner of the posterior hypothalamus box. For the parasubthalamic nucleus, we put a 400 × 200 µm box with the lateral border at the ventral edge of the cerebral peduncle 2.54 mm caudal to bregma. For the periaqueductal gray, we put a 600 × 700 µm oval box around the midbrain aqueduct. For the anterior pretectal nucleus, we placed a 400 × 500 µm box with the dorsomedial corner was 300 µm below and 1200 µm lateral to the dorsal edge of the aqueduct. The Edinger-Westphal nucleus can be easily identified by Cresyl violet staining and a 50 × 250 µm oval box was placed on it. For the ventral tegmental area, we placed a 400 × 400 µm box with the medial border at the lateral edge of the fornix 3.16 mm caudal to bregma. The locus coeruleus can be easily identified as a dense crescent moon-shaped region stained by Cresyl violet. We put an isosceles triangle of 500 × 250 µm (bottom x height) on it 5.52 mm caudal to bregma. To count cells in the nucleus of the solitary tract, we used a trapezoidal box (dorsal side: 200 µm; lateral side: 300 µm; ventral side; 400 µm) with the medial edge adjacent to the area postrema 7.48 mm caudal to bregma. For each of these regions, the anterior–posterior coordinates are from the Paxinos mouse brain atlas^49^. For each region, two sections from an animal were bilaterally counted and the average value was used as the value for the region in that animal. Cell counts were adjusted using the Abercombie correction factors^50^ and normalized as numbers in the unit area because the counting box sizes were largely different among the nuclei.

### Quantification of lesion size and viral spread

In experiment 2 (see below), lesion size was determined by lack of neuron-specific nuclear protein NeuN staining in the NAc. The outline of the lesioned area was marked in the images using Adobe Photoshop and the area size was measured using the measuring tool in the software. Three slices within 1.2–1.6 mm from bregma were selected per mouse and the areas lesioned were measured bilaterally. For better measurement, lesion size was normalized to total area of the NAc shell and expressed as a percentage.

In experiment 3 (see below), spread of AAV was determined by distribution of mCherry linked to hM4Di and hM3Dq receptors among the cells stained with a green fluorescent Nissl stain. The spread area of mCherry was marked in the image of the slice located 1.4 mm from bregma.

### Experimental design

#### Experiment 1: Identifying brain regions activated in association with chocolate-induced cataplexy

To examine brain regions activated specifically during chocolate-induced cataplexy, mice were quickly removed from the recording chamber when they showed behavior associated with cataplexy within 1 min of consuming chocolate (Fig. 1A). When we confirmed cataplexy through abrupt recovery of movement after the attack (~2 min), the mice were immediately anesthetized with urethane (1.8 g/kg) and transcardially perfused as described in the immunohistochemistry section. Perfusion was started within 5 min after the injection of anesthetic and after confirmation of adequate anesthesia by a lack of a response to toe pinch. The no cataplexy control was taken when the mice consumed chocolate and cataplexy did not occur within 3 min. In a third group, mice were anesthetized 5 s after the start of cataplexy to minimize the possible effect of cataplexy itself on other brain activities. Brain regions were systematically examined for expression of pERK as described in the quantitative analysis of histology section.

#### Experiment 2: Effect of chemical lesioning of NAc on cataplexy and tongue protrusion

Two weeks after injection of ibotenic acid or PBS into the NAc, animal behavior was continuously monitored for 12 hr without any disruption during the dark period. The animal was subjected to the tongue protrusion test after a 3–4 day interval. Finally, mice were deeply anesthetized with urethane and brains were collected as described in the immunohistochemistry section. Lesion size was determined through lack of NeuN staining in the NAc.

#### Experiment 3: Effect of activation/inactivation of NAc on cataplexy

Two weeks after injection of AAV or PBS into the NAc, cataplexy behavior was measured for 12 hr during the dark period (Fig. 3A). At 07:00 (start of the dark period), saline (0.03 ml/g) was injected i.p. and behavior was observed for the following 12 hr. After an interval of 3–4 days, CNO (0.45 mg/kg) was tested in a similar manner. One week after the completion of behavioral observation, the mice were subjected to the tongue protrusion test 1 hr after injection of saline or CNO (interval of 3–4 days). Finally, the mice were deeply anesthetized (90 min after administration of CNO) with urethane and brains were collected and analyzed as described in the immunohistochemistry section. Possible activation/inactivation of NAc neurons was examined using c-Fos but not pERK staining because expression of pERK is so transient^7^ that c-Fos may be more suitable for the relatively long action induced by CNO^25^. Another set of ORX-AB mice were injected with saline and euthanized 90 min after the injection to serve as the control for c-Fos expression in the NAc.

### Drugs

We used ibotenic acid (neurotoxin for AMPA/NMDA receptors, #14584, Cayman Chemical Company), Clozapine-N-oxide (CNO, C0832, Sigma), AAV(5)-CaMKIIα-hM3Dq-mCherry (50476-AAV5, 4.4 × 10^12^ GC/ml, addgene, Cambridge, MA, USA), and AAV(5)-CaMKIIα-hM4Di-mCherry (50477-AAV5, 3.1 × 10^12^ GC/ml, addgene). CNO was first dissolved in water (0.1 M) and diluted with saline just before use.

### Statistical analysis

Statistical analyses were performed using Prism software v.6 (Graphpad). For the data in experiment 1, we used a two-way ANOVA (sampling timing x brain regions) followed by Sidak’s multiple comparison test. For the data in experiment 2, we used a t-test and regression analysis. For the data in experiment 3, we used a two-way ANOVA (pretreatment in the NAc: PBS, AAV-Gi, or AAV-Gq x intraperitoneally injected drugs: saline or CNO) with a repeated measures design followed by Sidak’s multiple comparison test. For analysis of c-Fos numbers, we used a one-way ANOVA followed by Tukey’s multiple comparison test. P < 0.05 was considered statistically significant. All data are presented as mean ± standard error of the mean.

## References

[1] Suardi A, Sotgiu I, Costa T, Cauda F, Rusconi M (2016). The neural correlates of happiness: A review of PET and fMRI studies using autobiographical recall methods. Cogn. Affect. Behav. Neurosci..

[2] Berridge KC, Kringelbach ML (2015). Pleasure systems in the brain. Neuron.

[3] Krahn LE, Lymp JF, Moore WR, Slocumb N, Silber MH (2005). Characterizing the emotions that trigger cataplexy. J. Neuropsychiat Clin. Neurosci..

[4] Burgess CR, Oishi Y, Mochizuki T, Peever JH, Scammell TE (2013). Amygdala lesions reduce cataplexy in orexin knock-out mice. J. Neurosci..

[5] Chemelli RM (1999). Narcolepsy in orexin knockout mice: molecular genetics of sleep regulation. Cell.

[6] Oishi Y (2013). Role of the medial prefrontal cortex in cataplexy. J. Neurosci..

[7] Antoine B, Serge L, Jocelyne C (2014). Comparative dynamics of MAPK/ERK signalling components and immediate early genes in the hippocampus and amygdala following contextual fear conditioning and retrieval. Brain Struct. Funct..

[8] Ikoma Y, Kusumoto-Yoshida I, Yamanaka A, Ootsuka Y, Kuwaki T (2018). Inactivation of serotonergic neurons in the rostral medullary raphé attenuates stress-induced tachypnea and tachycardia in mice. Front. Physiol..

[9] Tabuchi S (2014). Conditional ablation of orexin/hypocretin neurons: A new mouse model for the study of narcolepsy and orexin system function. J. Neurosci..

[10] Thannickal T (2000). Reduced number of hypocretin neurons in human narcolepsy. Neuron.

[11] Saddoris MP, Cacciapaglia F, Wightman RM, Carelli RM (2015). Differential dopamine release dynamics in the nucleus accumbens core and shell reveal complementary signals for error prediction and incentive motivation. J. Neurosci..

[12] Calipari ES (2016). *In vivo* imaging identifies temporal signature of D1 and D2 medium spiny neurons in cocaine reward. PNAS.

[13] Nunes EJ, A. Randall P, Podurgiel S, Correa M, D. Salamone J (2013). Nucleus accumbens neurotransmission and effort-related choice behavior in food motivation: Effects of drugs acting on dopamine, adenosine, and muscarinic acetylcholine receptors. Neurosci. Biobehav. Rev..

[14] Ferrario CR (2016). Homeostasis Meets Motivation in the Battle to Control Food Intake. J. Neurosci..

[15] Wenzel JM, Rauscher NA, Cheer JF, Oleson EB (2015). A role for phasic dopamine release within the nucleus accumbens in encoding aversion: A review of the neurochemical literature. ACS Chem. Neurosci..

[16] Salamone, J. D. *et al*. In *Behavioral Neuroscience of Motivation. Current Topics in Behavioral Neurosciences, vol 27*. (eds. Simpson, E. & Balsam, P.) 231–257 (Springer, Cham, 2015). 10.1007/7854_2015_402 PMC4864984 26602246

[17] Corbit, L. H. & Balleine, B. W. In B*eha*vioral N*eu*rosci*ence of Motivation. Current Topics in Behavioral Neurosciences, vol 27*. (eds. Simpson, E. & Balsam, P.) 259–289 (Springer, Cham, 2015).

[18] Gold PW (2015). The organization of the stress system and its dysregulation in depressive illness. Mol. Psychiat.

[19] Francis TC, Lobo MK (2017). Emerging role for nucleus accumbens medium spiny neuron subtypes in depression. Biol. Psychiat.

[20] Murphy LO, Blenis J (2006). MAPK signal specificity: the right place at the right time. Trends Biochem. Sci..

[21] Chou TC (2001). Orexin (hypocretin) neurons contain dynorphin. J. Neurosci..

[22] Futatsuki, T. *et al*. Involvement of orexin neurons in fasting- and central adenosineinduced hypothermia. *Sci Rep* **8**, 10.1038/s41598-018-21252-w (2018). 10.1038/s41598-018-21252-w PMC5807529 29426934

[23] Groenewegen HJ, Wright CI, Beijer AVJ, Voorn P (2006). Convergence and segregation of ventral striatal inputs and outputs. Ann. NY. Acad. Sci..

[24] Ho C-Y, Berridge KC (2014). Excessive disgust caused by brain lesions or temporary inactivations: Mapping hotspots of nucleus accumbens and ventral pallidum. Eur. J. Neurosci..

[25] Gomez JL (2017). Chemogenetics revealed: DREADD occupancy and activation via converted clozapine. Sci..

[26] Butler T (2009). Clozapine-Induced Negative Myoclonus is not Cataplexy. J. Neuropsychiat Clin. Neurosci..

[27] Chiles J, Cohn S, McNaughton A (1990). Dropping objects: possible mild cataplexy associated with clozapine. J. Nerv. Ment. Dis..

[28] Subramanian S, Völlm B, Huband N (2017). Clozapine dose for schizophrenia. Cochrane Database Syst. Rev..

[29] Hasegawa E (2017). Serotonin neurons in the dorsal raphe mediate the anticataplectic action of orexin neurons by reducing amygdala activity. PNAS.

[30] Liu M, Blanco-Centurion C, Shiromani PJ (2017). Rewiring brain circuits to block cataplexy in murine models of narcolepsy. Curr. Opin. Neurobiol..

[31] Morawska M, Buchi M, Fendt M (2011). Narcoleptic episodes in orexin-deficient mice are increased by both attractive and aversive odors. Behav. Brain Res..

[32] Sakurai T (2007). The neural circuit of orexin (hypocretin): maintaining sleep and wakefulness. Nat. Rev. Neurosci..

[33] Ekman P (1993). Facial expression and emotion. Am. Psychol..

[34] Kaneko S (2000). Synaptic Integration Mediated by Striatal Cholinergic Interneurons in Basal Ganglia Function. Sci..

[35] Robison AJ (2013). Behavioral and structural responses to chronic cocaine require a feedforward loop involving FosB and calcium/calmodulin-dependent protein kinase II in the nucleus accumbens shell. J. Neurosci..

[36] Frey S, Morris R, Petrides M (1997). A neuroanatomical method to assess the integrity of fibers of passage following ibotenate-induced damage to the central nervous system. Neurosci. Res..

[37] Schwarcz R (1979). Ibotenic acid-induced neuronal degeneration: A morphological and neurochemical study. Exp. Brain Res..

[38] Shansky RM (2019). Are hormones a “female problem” for animal research?. Sci..

[39] Dijk D-J (2012). Imaging and monitoring sleep and its disorders: local sleep, circadian rhythms and variability. J. Sleep. Res..

[40] Miyata K, Kuwaki T, Ootsuka Y (2016). The integrated ultradian organization of behavior and physiology in mice and the contribution of orexin to the ultradian patterning. Neurosci..

[41] Scammell TE, Willie JT, Guilleminault C, Siegel JM, Narcolepsy IWGORMO (2009). A consensus definition of cataplexy in mouse models of narcolepsy. Sleep..

[42] Castro DC, Berridge KC (2014). Opioid hedonic hotspot in nucleus accumbens shell: Mu, delta, and kappa maps for enhancement of sweetness “Liking” and “Wanting”. J. Neurosci..

[43] Tappaz M, Brownstein M, Palkovits M (1976). Distribution of glutamate decarboxylase in discrete brain nuclei. Brain Res..

[44] Benson DL, Isackson PJ, Hendry SHC, Jones EG (1991). Differential gene expression for glutamic acid decarboxylase and type II calcium-calmodulin dependent protein kinase in basal ganglia, thalamus, and hypothalamus of the monkey. J. Neurosci..

[45] Wang X, Zhang C, Szábo G, Sun Q-Q (2013). Distribution of CaMKIIα expression in the brain *in vivo*, studied by CaMKIIα-GFP mice. Brain Res..

[46] Wang JQ, Fibuch EE, Mao L (2007). Regulation of mitogen-activated protein kinases by glutamate receptors. J. Neurochem..

[47] Ortiz J (1995). Extracellular signal-regulated protein kinases (ERKs) and ERK kinase (MEK) in brain: Regional distribution and regulation by chronic morphine. J. Neurosci..

[48] Papa M, Sergeant JA, Sadile AG (1998). Reduced transduction mechanisms in the anterior accumbal interface of an animal model of attention-deficit hyperactivity disorder. Behav. Brain Res..

[49] Paxinos, G. & Franklin, K. B. J. *The Mouse Brain in Stereotaxic Coordinates*. (Academic Press, 2001).

[50] Guillery RW (2002). On counting and counting errors. J. Comp. Neurol..

